# A Perspective on Polylactic Acid-Based Polymers Use for Nanoparticles Synthesis and Applications

**DOI:** 10.3389/fbioe.2019.00259

**Published:** 2019-10-11

**Authors:** Tommaso Casalini, Filippo Rossi, Andrea Castrovinci, Giuseppe Perale

**Affiliations:** ^1^Polymer Engineering Laboratory, Department of Innovative Technologies, Institute for Mechanical Engineering and Materials Technology, University of Applied Sciences of Southern Switzerland, Manno, Switzerland; ^2^Department of Chemistry, Materials and Chemical Engineering “Giulio Natta”, Politecnico di Milano, Milan, Italy; ^3^Ludwig Boltzmann Institute for Experimental and Clinical Traumatology, Vienna, Austria

**Keywords:** polylactic acid, degradation, processing, nanomedicine, nanoparticles

## Abstract

Polylactic acid (PLA)—based polymers are ubiquitous in the biomedical field thanks to their combination of attractive peculiarities: biocompatibility (degradation products do not elicit critical responses and are easily metabolized by the body), hydrolytic degradation *in situ*, tailorable properties, and well-established processing technologies. This led to the development of several applications, such as bone fixation screws, bioresorbable suture threads, and stent coating, just to name a few. Nanomedicine could not be unconcerned by PLA-based materials as well, where their use for the synthesis of nanocarriers for the targeted delivery of hydrophobic drugs emerged as a new promising application. The purpose of the here presented review is two-fold: on one side, it aims at providing a broad overview of PLA-based materials and their properties, which allow them gaining a leading role in the biomedical field; on the other side, it offers a specific focus on their recent use in nanomedicine, highlighting opportunities and perspectives.

## Introduction

Polylactic acid (PLA), classified as an aliphatic polyester because of the ester bonds that connect the monomer units, has gained a key role in the biomedical field for a wide range of applications: suture threads, bone fixation screws, devices for drug delivery, just to scratch the surface. PLA merges several interesting properties that make it an ideal candidate for biomedical applications.

PLA naturally degrades *in situ* through hydrolysis mechanism: water molecules break the ester bonds that constitute polymer backbone. This eliminates the necessity of additional surgeries in order to remove the device, improving patient recovery and optimizing health system costs.

The main phenomena involved in the degradation mechanisms and the most important factors that influence hydrolysis rate are currently well-established in scientific literature, thanks to a devoted research activity that reached the peak between the 1980s and the 1990s. Consequently, degradation kinetics and mechanical properties can be tailored by properly tuning few polymer properties (such as composition or molecular weight), thus leading to the development of biomedical devices optimized for each specific application. Degradation products (composed of lactic acid and its short oligomers) are recognized and metabolized by the body itself: this gives PLA an intrinsic biocompatibility that dampens the attainment of critical immune responses. In addition, PLA can be processed with standard and established technologies, such as injection molding, extrusion, etc.

After this brief summary, whose main points will be discussed in the following sections, it should be no more surprising why PLA attracted a lot of attention and enthusiasm in the biomedical field. These features make PLA a suitable option also for the new paradigm recently introduced by nanomedicine, where nanomaterials (whose size is similar to molecules of biological interest, such as proteins or viruses) are distributed within the human body and can be internalized by cells.

Nanomedicine offers new unprecedented chances, thanks to the synthesis of nanoparticles, which can be employed for the targeted delivery of drugs, vaccines and genes. On the other hand, nanomaterials can also give rise to new side effects due to specific interactions with the biological components (proteins, carbohydrates, lipids) present in body fluids (blood, plasma, interstitial fluids).

The first part of this review guides the interested reader through the main peculiarities of PLA, underlining the reasons why it emerged as a material of choice in the biomedical field. The second part of the manuscript is focused on the use of PLA for the synthesis and application of nanoparticles, from the synthetic routes of nanovectors to perspectives and opportunities.

## Polylactic Acid-Based Materials: General Description and Synthesis Routes

**Figure 1:**
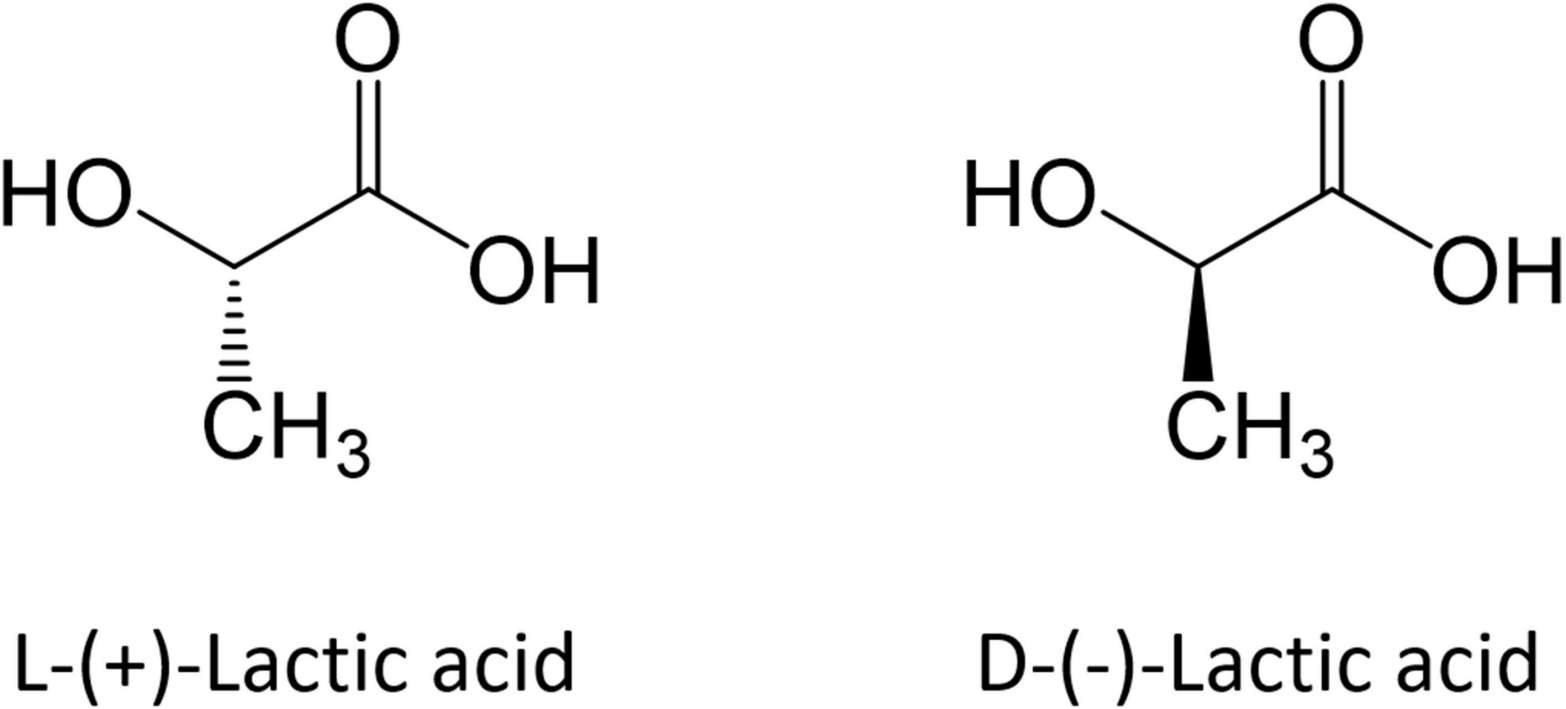
Enantiomeric forms of lactic acid.

Polylactic acid is a hydrophobic polymer that belongs to the class of biomaterials commonly referred as poly-α-hydroxy acids, poly-α-esters or aliphatic polyesters. It is synthesized starting from lactic acid (LA; 2-hydroxypropanoic acid), which a water-soluble monomer that exhibits two enantiomeric forms, namely L-(+)-LA and D-(-)-LA, as shown in Figure 1.

Although both enantiomers are employed in industrial practice, L-(+)-LA is the isomer of interest for biomedical applications since it is involved in the cellular metabolism of the human body and reduces the risk of adverse reactions. In *in vivo* environment L-(+)-LA can be either incorporated into the Krebs' cycle or converted into glycogen in the liver; eventually it is eliminated as water and carbon dioxide from the lungs (Sheikh et al., 2015). PLA can be produced starting from pure L-lactic and D-lactic isomers, which leads to poly-L-lactic (PLLA) acid and poly-D-lactic acid (PDLA) homopolymers, respectively; if a racemic mixture of L- and D-monomers is employed, poly-D,L-lactic acid (PDLLA) copolymer is obtained. The stereochemistry has a relevant impact on material properties: PLLA is a semi-crystalline polymer, while PDLLA is an amorphous polymer with no melting point. In addition, degradation rate of PLLA is significantly slower than PDLLA, because of the presence of crystalline regions. Main advantages and disadvantages of PLA use and production are summarized in Table 1.

**Table 1:** Main advantages and disadvantages of PLA.

**Advantages**	**Disadvantages**
**Eco-friendliness:** PLA is produced from renewable sources (corn, wheat, rice). In addition, it is biodegradable, recyclable and compostable. Its production consumes CO_2_.	**Poor thoughness:** PLA is a very brittle material, whose elongation at break is <10%. This can represent a limit for those applications that need plastic deformation at high stress levels.
**Biocompatibility:** main PLA degradation product, lactic acid, is non-toxic and metabolized by the organism itself.	**Slow degradation rate:** PLA naturally degrades through hydrolysis, whose rate depends on may factors, such as crystallinity and molecular weight. Slow PLA degradation leads to high life time of devices *in vivo*, and can raise issues for the disposal of commodities.
**Processability:** PLA has a better thermal processability than other biopolymers. It can be processed through injection molding, film extrusion, blow molding, thermoforming, fiber spinning, and film forming.	**Hydrophobicity:** PLA is a relatively hydrophobic material (static water contact angle value is about 80°). This results in low cell affinity and can lead to inflammatory response upon direct contact to biological fluids.
**Energy saving:** PLA requires 25–55% less energy than petroleum-based polymers.	**Lack of reactive side chain groups:** PLA is chemically inert, which makes surface functionalization and bulk modification challenging tasks.

*Adapted from Farah et al. (2016)*.

Focusing on lactic acid itself, synthesis can be performed in different ways; the most popular route is the following one (Storti and Lattuada, 2017):

(1) $$CH_{3}CHO+HCN\to CH_{3}CH\left(OH\right)CN$$

(2) $$\begin{array}{l} CH_{3}CH\left(OH\right)CN+2H_{2}O+HCl\to CH_{3}CH\left(OH\right)COOH\\ \;+NH_{4}Cl \end{array}$$

(3) $$\begin{array}{l} CH_{3}CH\left(OH\right)COOH+CH_{3}OH↔CH_{3}CH\left(OH\right)COOCH_{3}\\ \;+H_{2}O \end{array}$$

Lactonitrile, obtained from acetaldehyde and hydrogen cyanide (1), is hydrolysed at low pH in order to lactic acid (2); it is subsequently converted to methyl lactate (3) through esterification and eventually recovered and purified by distillation. Lactic acid and methanol are obtained through hydrolysis from lactate; methanol is recycled in step (3). Anyway, this kinetic pathway leads to a racemic mixture.

Bacterial fermentation of sugar solutions is currently the most employed process; this process leads to high yields and, depending on the chosen type of bacteria, it allows obtaining one given stereoisomer or the racemic mixture. It is estimated that about 90% of the total LA produced worldwide is currently obtained with this procedure.

In this framework, the critical step is the subsequent LA purification, which is expensive and determines process profitability. Commonly used techniques are liquid extraction, membrane separation, ion exchange, electrodialysis, and reactive distillation.

Polymer synthesis can be carried out through step growth polymerization or ring opening polymerization. Step growth polymerization simply takes advantage of the reactivity of the two LA functional groups: indeed, the polycondensation of hydroxyl and carboxyl moieties leads to the formation of the ester bonds that constitute polymer backbone. This synthetic route has several drawbacks: long residence times are required for longer chains (leading to unwanted side reactions, like transesterification), challenging reaction conditions (temperatures up to 250°C and vacuum up to 100 mbar) and continuous water (side product of polycondensation) removal. Chain extenders (e.g., isocyanates or epoxides) can be in principle employed, although this approach has an inevitable impact on material purity and quality.

**Figure 2:**
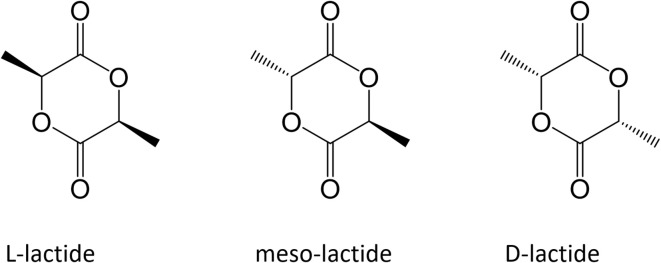
Cyclic dimers for ROP process.

At industrial scale ROP is the most popular process because of its advantages: mild process conditions, short residence times, absence of side products and high molecular weights. The most widely used catalyst is 2-ethylhexanoic tin(II) salt (also referred as stannous octoate [Sn(Oct)_2_]), approved by United Stated Food and Drug Administration (FDA) and usually employed along with an alcohol as cocatalyst. The real bottleneck of ROP is the availability of cyclic monomers as well as their optical and chemical purity, since impurities have detrimental effects on material properties due to the sensitivity of the reaction to residual non-cyclic monomers. The cyclic raw material for PLA is constituted by cyclic dimer lactide, which exhibits three stereoisomeric forms, as shown in Figure 2: LL-, DD-, and D,L-(also referred a meso-lactide).

**Figure 3:**
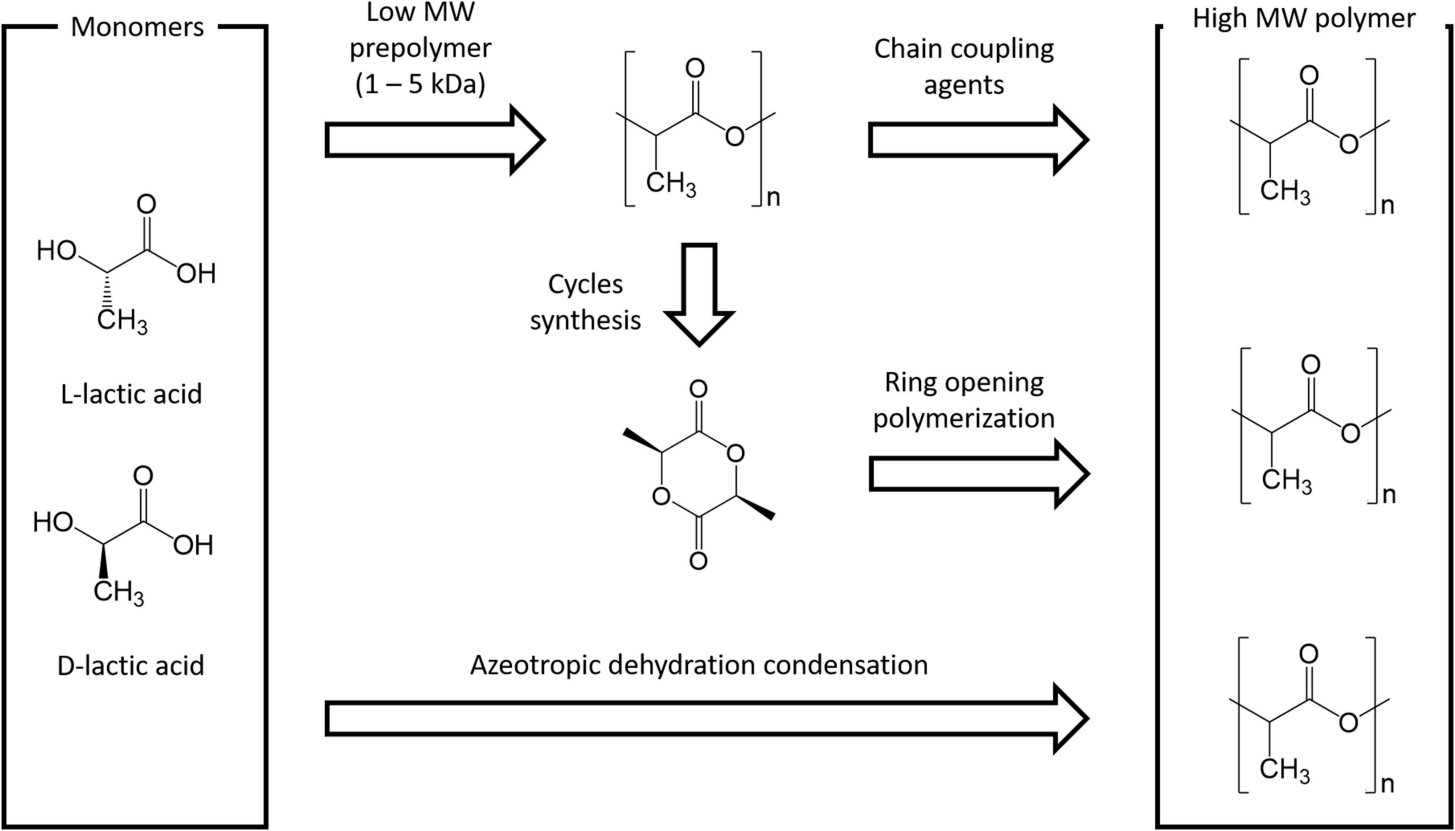
Main PLA production routes.

Lactide is usually produced through backbiting kinetic mechanism is then (promoted with suitable process conditions) starting from low molecular weight prepolymer; cycles are eventually collected by distillation. Other synthesis routes are azeotropic dehydration and enzymatic polymerization. PLA-based polymers synthesis routes are summarized in Figure 3.

PLA is widely employed in the biomedical field because of its biocompatibility and its processability, since it can be processed with a wide range of techniques, such as extrusion, injection molding, injection stretch blow molding, film and sheet casting, extrusion blow film, thermoforming, foaming, fiber spinning, electro spinning, blending, compounding, and nanocompositing.

## Physical and Chemical Properties

PLA can be seen as a “family” of polymers, which include homopolymers PLLA and PDLA (synthesized from mixtures of pure L- or D-lactic acid) and the copolymer PDLLA (obtained from the racemic mixture). This has a remarkable impact on material properties because of the involved stereochemistry: PLLA and PDLA are semicrystalline polymers, while PDLLA is usually amorphous. The final crystallinity depends also on the thermal and mechanical history, mainly due to fabrication processes. Mechanical properties are summarized in Table 2; values are expressed as ranges, since they strongly depend on the characteristic of the tested material (molecular weight, crystallinity, processing, etc.) as well as testing procedure (Van De Velde and Kiekens, 2002).

**Table 2:** Mechanical properties of PLA-based polymers.

**Property**	**PLA**	**PLLA**	**PDLLA**
ρ [g cm^−3^]	1.21–1.25	1.24–1.30	1.25–1.27
σ [MPa]	21–60	15.5–150	27.6–50
E [GPa]	0.35–0.5	2.7–4.14	1–3.45
ε [%]	2.5–6	3.0–10.0	2.0–10.0
T_g_ [°C]	45–60	55–65	50–60
T_m_ [°C]	150–162	170–200	amorphous–no melt point

*ρ, density; σ, tensile strength; E, elastic modulus; ε, ultimate strain; T_g_, glass transition temperature; T_m_, melting temperature. Taken from Farah et al. (2016)*.

Polymer crystallinity influences mechanical and physical properties such as hardness, modulus, tensile strength, stiffness, and melting points. If the amount of PLLA is higher than 90% the polymer is semicrystalline, while lower amounts (and thus a lower optical purity) lad to an amorphous polymer. The density values lie in small range depending on the composition, as shown in Table 2.

PLA is soluble in dioxane, acetonitrile, chloroform, methylene chloride, 1,1,2-trichloroethane and dichloroacetic acid, while it is only partially soluble in ethyl benzene, toluene, acetone and tetrahydrofuran, only when heated to boiling temperature. PLA is not soluble in water, alcohols, and linear hydrocarbons. Crystalline PLLA cannot be dissolved in acetone, ethyl acetate, or tetrahydrofuran.

It is worth mentioning that polymer properties can change after processing, because of thermal and mechanical stresses. PLA undergoes thermal degradation above 200°C, although degradation rate and extent depend on variables like time, temperature, low molecular weight impurities, and catalyst amount (Carrasco et al., 2010).

The success of PLA passes also through its versatility, since material properties can be modified in several ways. They can be tuned, e.g., through the addition of suitable plasticizers, widely used in order to improve processability and flexibility of polymers. Focusing on semicrystalline PLA, plasticizer addition decreases *T*_*g*_, as well as *T*_*m*_ and crystallinity.

PLA can be blended with biodegradable or non-biodegradable polymers (such as polyethylene, polypropylene, chitosan, polystyrene, polyethylene terephthalate, and polycarbonates) (Saini et al., 2016) or by making composite materials (Murariu and Dubois, 2016) through the addition of carbon nanotubes, ceramic nanoparticles, natural fibers, and cellulose (Hamad et al., 2018). A relevant example is constituted by PLA/hydroxyapatite (HA) composites for devices for bone healing, where HA micro or nanoparticles are dispersed into the polymer matrix (Rodenas-Rochina et al., 2015).

Another is the formation of stereocomplexes (Tsuji, 2016), which can be obtained by blending PLLA with PDLA (that is, the homopolymer composed by D-lactide units only) or adopting PLLA/PDLA block copolymers. The strong interactions between PDLA and PLLA blocks that derive from the formation of stereocomplex crystallization improves mechanical properties and thermal stability, slows down degradation rate and increase PLA barrier properties, allowing a more prolonged drug release. PLA-based stereocomplexed materials, by virtue of their improved stability, attracted a lot of interest also for biomedical applications, such as fibers and nanoparticles for drug delivery applications (Jing et al., 2016).

PLA-based materials can be also assembled in complex molecular architectures (Corneillie and Smet, 2015), leading to branched polymer chains, star-shaped structures (Michalski et al., 2019), grafted chains (Nagahama et al., 2007), and cross-linked matrices (Tsuji, 2016). If synthesized with both PLLA and PDLA blocks, stereocomplexation can be achieved also with these complex structures (Nagahama et al., 2007; Fan et al., 2013; Sveinbjornsson et al., 2014), which found as well-potential applications in the biomedical field for the synthesis of hydrogels, nanoparticles and micelles for drug delivery purposes.

Another popular way to tune material properties is the copolymerization with glycolic acid, which leads to the well-known polylactic-co-glycolic random copolymer (PLGA). Copolymerization is also performed with caprolactone, which allows obtaining polylactic-co-caprolactone (PLCL). Another strategy to improve material hydrophilicity is the synthesis of PLA and polyethylene glycol (PEG) block copolymers.

PLA (as well as its copolymers) degrades because of hydrolysis mechanism: water breaks the ester bonds that constitute polymer backbone, according to the following mechanism:

(4) $$\begin{array}{r} P_{n+m}+H_{2}O+H^{+}↔P_{n}+P_{m}+H^{+} \end{array}$$

where *P*_*n*+*m*_, *P*_*n*_, and *P*_*m*_ are polymer chains composed by *n*+*m, n*, and *m* monomer units, respectively, *H*_2_*O* is a water molecule and *H*^+^ indicates that hydrolysis is catalyzed in acidic environment. The most important phenomena that govern PLA degradation are currently rationalized and accepted in scientific literature (Casalini, 2017). Two degradation regime can be distinguished. If hydrolysis rate is higher than diffusion rate, surface, or heterogeneous degradation takes place; only polymer surface experiences degradation and erosion (i.e., mass loss), while the bulk remains intact. The shape of the device remains unchanged, but its volume decreases in time. On the other hand, if water penetration is much faster than water consumption, homogeneous, or bulk degradation occurs: degradation rate is essentially equal in every point of the matrix and the volume does not appreciably change in time. Mass loss is observed after a certain time interval, when chain scission has created oligomers that are mobile enough to diffuse through the matrix toward the environment. Another relevant aspect is autocatalysis: polymer degradation creates small fragment that lower pH-value by virtue of their dissociated carboxyl terminal group, thus enhancing hydrolysis rate. In other words, pH decreases as degradation continues and this results in an autocatalytic behavior. Notably, when mass transport resistances and/or mean diffusive paths are relevant, a transition from homogeneous to heterogeneous degradation may occur. In this case, oligomers accumulate in the core of the device, locally lowering the pH; consequently, degradation is faster in the bulk than close to the surface. In order to discriminate the degradation mechanism, Von Burkersroda et al. (2002) proposed a distinctive parameter called critical thickness *L*_*crit*_; if the characteristic size of the device (e.g., the radius of a sphere) is larger than the critical thickness surface degradation occurs, otherwise bulk degradation govern matrix hydrolysis mechanism.

**Figure 4:**
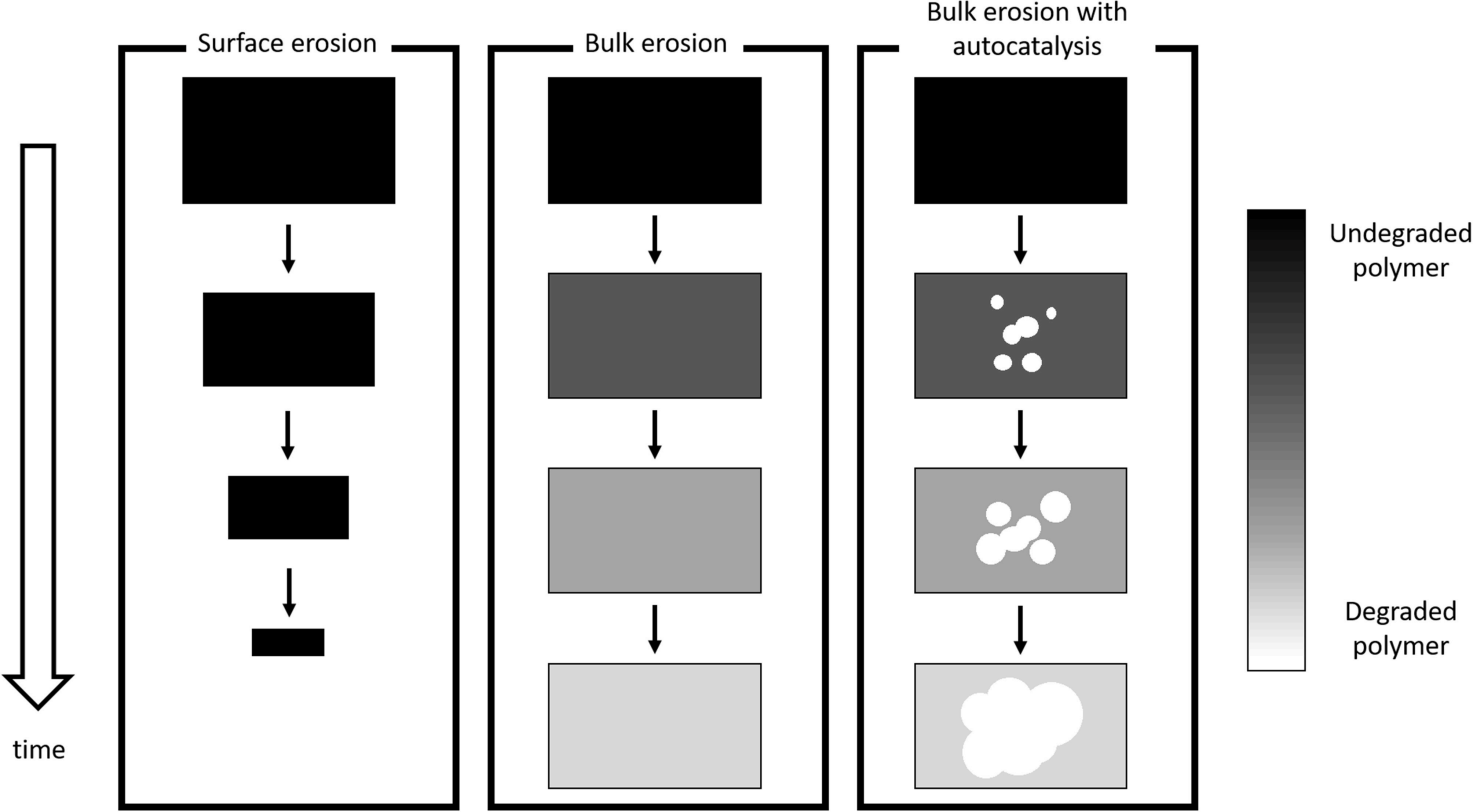
Schematic of degradation mechanisms.

A scheme is provided in Figure 4.

The discussed mechanisms represent asymptotic cases and the observed experimental behavior is usually one of the many shades of gray in between.

*L*_*crit*_ depends on the interplay between degradation and diffusion kinetics, and at a first glance, it depends on the specific material. Von Burkersroda et al. (2002) computed the values of *L*_*crit*_ for some polymers of interest; the reference value for aliphatic polyesters is 7.4 cm. The main phenomena behind PLA degradation can be summarized as follows (Casalini, 2017):

- Water penetrates into the polymer matrix from the surrounding environment through diffusion. PLA is hydrophobic and polymer dissolution is absent; volumetric swelling is negligible;
- While water diffuses, it breaks the ester bonds and causes chain scission;
- The resulting small oligomers diffuse out of the matrix; if their concentration in the core is high because of mass transport resistances, degradation is locally faster due to autocatalysis;
- Diffusivities of each compound increase as molecular weight decreases, since chain scission creates new and wider diffusive paths;
- In *in vivo* environment, an additional contribution to degradation is given by enzymes, which contribute to the erosion of device surface (*vide infra*).

Degradation rate depends on several factors, such as (Alexis, 2005):

- *Polymer composition*: Generally speaking, degradation increases as material hydrophilicity increases. PLLA degradation is slower than PDLLA because of the presence of crystalline regions.
- *pH*: As mentioned, hydrolysis of ester bond is favored at low pH-values, although there are some experimental evidences that basic conditions can speed up chain scission.
- *Device geometry*: Device size can discriminate the attainment of bulk or surface degradation (*vide supra*).
- *Molecular weight*: Degradation rate decreases as molecular weight increases, because of the lower water uptake. In addition, high molecular weight values imply a lower concentration of carboxyl end groups.
- *Crystallinity*: Broadly, semicrystalline polymers are characterized by a slower degradation rate than amorphous ones, since crystallites regions are less subjected to hydrolysis. However, there are still some inconsistent results, which may depend on the different process methodologies. It has also been observed that the short chains that derive from the degradation of amorphous regions gain enough mobility to organize themselves in crystalline regions.
- *Addition of drugs and/or additives*: The addition of acidic compounds can enhance hydrolysis rate, while basic compounds can neutralize carboxyl end groups and enhance degradation through base catalysis. The addition of plasticizers can promote water diffusion and water uptake, enhancing degradation rate.
- *Sterilization*: The use of beta or gamma irradiation or sterilization results in undesired reactions such as chain scissions and cyclization, that lower molecular weight and enhance degradation rate.
- *Mechanical stress*: Stress fields due to specific applications (e.g., fixation screw) enhance degradation rate.
- *Fabrication processing*: Thermal and mechanical stresses experienced by the polymer during commonly employed processing techniques (extrusion, injection molding, etc.) can lead to decrease in molecular weight and increase of degradation rate.

By virtue of a critical thickness equal to 7.4 cm, PLA-based devices usually experience homogeneous degradation, which can become heterogeneous when oligomer accumulation in the bulk occurs.

In *in vivo* environment, there is an additional contribution to degradation due to enzymes that cleave ester bonds, such as lipases, cutinases, serine proteases, PHB depolymerase, PCL depolymerase, elastase esterase, proteinase K, and trypsin. This enzymatic degradation is a heterogeneous process since involves only device surface: enzymes are not able to diffuse in the polymer matrix and contribute to surface erosion through ester bonds cleavage (Armentano et al., 2018).

PLA-based materials are also subjected to thermal degradation; while this is not relevant for biomedical applications themselves (at body temperature, thermal degradation is absent), this should be taken into account in the fabrication process. Indeed, for temperature values above 200°C (Garlotta, 2001), PLA experiences not only hydrolysis but also lactide reformation, oxidative chain scission, intra- or intermolecular transesterification reactions.

## Processes for Nanoparticles Synthesis

PLA-based materials experienced a wide success in biomedical field for several reasons: biocompatibility, low toxicity, degradation through hydrolysis and tailored physical and chemical properties through the selection of molecular weight or through copolymerization, blending, or building more complex molecular architectures and processability. A proper tuning of polymer properties allows assuring the desired performances (in terms, e.g., of tensile strength or release rate) over a suitable time span, before an appreciable onset of degradation reactions. The natural degradation of PLA-based devices due to hydrolysis avoids the need of additional surgery for device removal, improving patient care.

All these advantages led to a wide range of applications, summarized in Table 3 for the sake of completeness.

**Table 3:** Overview of PLA biomedical applications.

**Field**	**Application**
Orthopedic	Peripheral nerve and spinal cord injury regeneration Bioadsorbable screws Meniscus repair Guided bone regeneration
Cardiac	Chest wall reconstruction Stent
Dentistry	Guided tissue regeneration Biocompatible space fillers
Plastic surgery	Suture Reconstructive surgery Dermal fillers Skin draft
General surgery	Hemia mesh
Gynecology	Stress incontinence mesh
Radiology	Theranostic imaging
Oncology	Nanoparticles for drug delivery

*Adapted from Tyler et al. (2016)*.

Nanomedicine is an emerging field, focused on the development and application of engineered nanomaterials, whose size (from 1 to 1,000 nm according to the FDA draft guideline form 2017) is comparable to many molecules of biological interest, such as proteins and viruses. Devices like polymer nanoparticles, by virtue of their small size, can be internalized by cells and this opens a wide range of new opportunities for the development, e.g., of new carriers for the targeted delivery of drugs and vaccines or image contrast agents for diagnostic purposes. Because of the interesting properties of PLA-based polymers, it is not surprising that they experienced and are still experiencing a great interest as starting materials for the synthesis of nanoparticles.

The most frequently used and promising methods to formulate nanosized particles can be divided in four categories according to the fundamental physical principles, as summarized by Lee et al. (2016). The main challenges are the control of particle size and an efficient drug encapsulation.

### Emulsion-Based Methods

The single-emulsion/solvent-extraction method is the simplest approach for the synthesis of micro- and nanoparticles, including drug-loaded carriers. The polymer and, if needed, the hydrophobic drug are dissolved in a water-immiscible organic solvent and an emulsion in water phase is subsequently realized by adding a stabilizer and stirring. For the sake of completeness, oil in water (o/w) as well as oil in oil (o/o) and water in oil (w/o) emulsions can be suitable for this process.

The removal of the organic phase is carried out through evaporation at low pressure or vacuum or by solvent extraction; polymer particles are recovered by centrifugation or filtration and washed with water or buffer solutions in order to remove possible traces of solvent, stabilizer and free drug before lyophilization.

Single-emulsion approach leads to a poor encapsulation efficiency of hydrophilic drugs (such as peptides), which are mainly dispersed in the aqueous phase rather than the organic one. Double-emulsions methods aim at overcoming this issue. A water solution containing the hydrophilic active molecule is added to an organic solvent where the polymer is dissolved under stirring, in order to form a w/o (or an o/w) emulsion that is subsequently added to a second water phase containing a stabilizer. This leads to the formation of a w/o/w (or an o/w/o) emulsion. The organic solvent is removed by means of evaporation under low pressure or vacuum and the resulting particles are washed (to safely remove traces of solvent, stabilizer, and free drug) before lyophilization.

Emulsion-based methods, despite their simplicity, need the optimization of several process parameters, such as phase volumes (oil and water), polymer, drug and stabilizer concentration, type of solvents, and stirring rate.

Examples of particles produced with emulsion-based methods are provided in Table 4.

**Table 4:** Examples of nanoparticles synthesis by means of emulsion-based methods.

**Loaded drug**	**Preparation method**	**Particle size [nm]**	**References**
Bovine Serum Albumin	Double emulsion	140–250	Gao et al., 2005
Nimesulide	Emulsion-solvent evaporation	160–2,150	Freitas and Marchetti, 2005
Tetanus toxoid	Double emulsion	353–1,153	Bilati et al., 2005
Lysozyme	Double emulsion	369–459	Bilati et al., 2005
Insulin	Double emulsion	1,000–1,400	Bilati et al., 2005
Betamethasone phosphate	O/w emulsion	90–250	Ishihara et al., 2005
Vanillin	O/w emulsion	240	Dalmolin et al., 2016
Hemoglobin	Double emulsion	122–185	Sheng et al., 2009
Neurotoxin-I	Double emulsion	65	Cheng et al., 2008
Triclosan	Double emulsion	207–286	Pinon-Segundo et al., 2005
Paclitaxel	Single emulsion	110	Feng et al., 2015

### Precipitation-Based Methods

Nanoprecipitation, also referred as solvent displacement, is a one-step process suitable for producing nanoparticles loaded with hydrophobic drugs. The underlying physical principle is the interfacial deposition of a polymer, following the displacement of a water-compatible solvent from a lipophilic solution. Polymer and drug are dissolved in a semi-polar organic solvent (acetone, methanol, ethanol, acetonitrile); the resulting organic phase is mixed drop-wise in a water solution containing a stabilizer. This technique leads a narrow particle size distribution and allows avoiding the use of large amounts of toxic solvents as well as external energy sources. On the other hand, it is limited by drug solubility in the organic phase and it is thus not suitable for hydrophilic drugs; another drawback is the removal of the residual solvent.

Salting-out method is based on the addition of a polymer and drug solution in a water-compatible solvent (acetone, acetonitrile, tetrahydrofuran) to an aqueous solution that contains the salting-out agent (electrolytes like magnesium chloride and calcium chloride or non-electrolytes like sucrose) and a stabilizer, under stirring. This allows obtaining an o/w emulsion that is subsequently diluted with large volumes of water, promoting the formation of particles by virtue of the diffusion of the water-compatible solvent toward the aqueous phase. Particles are recovered and purified by means of centrifugation or filtration. In particular, salt residues must be removed before utilization.

Salting-out is the ideal process for encapsulating heat-sensitive molecules (such as proteins, DNA, RNA) because no heating steps are required. Anyway, the process requires the optimization of parameters like salt type and concentration, type of polymer and solvent, and their relative amounts. The principal limitations are the intensive purification of the resulting nanoparticles as well as incompatibility issues concerning most of the employed salts with bioactive compounds.

Dialysis emerged as a simple process that allows obtaining small particles with a narrow size distribution. The polymer is dissolved in an organic solvent and placed in a dialysis tube of suitable pore size; dialysis is subsequently carried out in a solvent that is miscible with the organic phase but not compatible with the polymer. This leads to the formation of polymer particles due to the loss in solubility. Selected examples from literature of particles synthesized by means of precipitation based-methods are reported in Table 5.

**Table 5:** Examples of nanoparticles synthesis by means of precipitation-based methods.

**Loaded drug**	**Preparation method**	**Particle size [nm]**	**References**
Sodium cromoglycate	Nanoprecipitation	470–1,300	Peltonen et al., 2004
Lysozyme	Nanoprecipitation	137–351	Bilati et al., 2005
Tyrphostin	Nanoprecipitation	65–143	Chorny et al., 2002
Cloricromene	Nanoprecipitation	120–340	Leo et al., 2004
–	Nanoprecipitation	100–300	Legrand et al., 2007
–	Salting out	100–400	Zweers et al., 2003
–	Salting out	279	Nguyen et al., 2003
–	Salting out	248	Zweers et al., 2004
Savoxepine	Salting out	274–736	Leroux et al., 1996
–	Dialysis	40–250	Lo et al., 2005
Epirubicin	Dialysis	128–1,088	Liu et al., 2007
Paclitaxel	Dialysis	367–475	Zhang et al., 2008
HIV p24 protein	Dialysis	200	Aline et al., 2009

### Compositing Methods

In spray drying technique, polymer and drug are dissolved in an organic solvent and subsequently dispersed as ultra-fine droplets in a hot air flow. The solvent evaporates instantaneously and dried particles are collected under low pressure in dry air flow. Spray drying process is easy to perform and can be potentially employed at industrial scale. However, productivity can be hindered by the adhesion of the particles to the walls of the spray dryer and their agglomeration. Moreover, it is challenging to control drug distribution.

Melting technique allows avoiding the use of organic solvents but implies the dissolution of the drug in a polymer melt; therefore, it is not suitable for encapsulating active compound that are subjected to thermal degradation. Drug/polymer melt is subsequently solidified and cooled down with water or dry air. Particles are obtained through grounding or milling; in order to achieve small particles with narrower size distribution, the ground melt can be emulsified in a hot solution with a stabilizer. Despite the absence of organic solvents, this approach is limited by the thermal treatment of the drug/polymer system and the high number of steps needed to obtain smooth particles.

*In situ*-forming techniques aim at overcoming the most common drawbacks of the discussed processes, such as solvent removal, particles recovering, and resuspension. A drug/polymer solution (in a water-miscible solvent) is prepared and injected in the target site. When in contact with physiological fluids, polymer phase hardens and precipitates forming microparticles that entrap the active compound. The main drawback lies in a careful choice of the solvent, whose side-effects must be previously investigated.

### Other Approaches

Supercritical fluids-based methods attracted a lot of interest because of their advantages, such as the use of environmentally friendly solvent and the possibility to obtain nanoparticles with (virtually) no traces of residual solvents. There are two main processes that involve supercritical fluids: rapid expansion of supercritical solution (RESS) and rapid expansion of a supercritical solution into a liquid solvent (RESOLV).

In RESS technique, the polymer is dissolved into the supercritical fluid; the solution is then subjected to a rapid expansion across a nozzle in ambient air. The sudden reduction in pressure leads to a substantial supersaturation, which, in turn, promotes homogeneous nucleation and the formation of well-dispersed particles. RESOLV process is based on the same principle, but the expansion does not take place in air but in a liquid solvent. The liquid phase hinders particle growth, leading to the synthesis of nanoparticles. The most important limitation of RESS and RESOLV technique is the poor solubility of the polymer in the supercritical fluid. In addition, it is difficult to control particle size and morphology.

With microfluidic techniques it is possible to obtain uniform particles with a narrow particle size distribution, which, in turn, allows a finer control of the release rate. The starting point is usually the attainment of an o/w emulsion in the microfluidic device, where monodisperse droplets can be achieved, followed by droplet solidification by means of solvent evaporation, diffusion or extraction. Particle size can be controlled by tuning the properties of oil and water phases (density, interfacial tension, and viscosity) and flow rates. Because of the inherent micron length scale, the challenge lies in the synthesis of particles at nanoscale. The underlying principle of hydrogel template method is the possibility to control sol-gel transition of physical gel by changing the environmental conditions (e.g., temperature). A warm aqueous gelling solution is distributed on a hard master template and placed at low temperature, in order to obtain a hydrogel mold. Polymer and drug are dissolved in a suitable solvent and poured on the hydrogel mold; solvent is removed through evaporation and particles are recovered by centrifugation or filtration and washed after dissolving the mold in water. Polyvinyl alcohol water-soluble molds are also employed. Similarly to microfluidic techniques, the drawback lies in the particle size, which is still limited to the micron length scale. In principle, nanoparticles can be obtained by means of nanostructured mold templates. Notably, nanoparticles can be produced not only from preformed polymers but also starting from monomers, including the polymerization process in the nanoparticle production step. This can be achieved my means of emulsion polymerization (George et al., 2019).

### Summary

For the sake of completeness, the main advantages and disadvantages of the most common employed methods are summarized in Table 6.

**Table 6:** Advantages and disadvantages of the most common nanoparticles production methods.

**Process**	**Advantages**	**Disadvantages**
Single/double emulsion	Particle size can be tuned acting on several variables (Table 7)	High shear rate High volumes of water to be removed
Nanoprecipitation	Nanoparticles have a well-defined size and a narrow size distribution Less toxic solvents No use of external energy sources	Extensive optimization of polymer/solvent/non solvent system Not suitable only for hydrophilic compounds
Salting out	No heating process required No hazardous/chlorinated solvents are employed	Extensive optimization of process conditions (type of salt and its concentration, type of polymer and solvent, and their ratio) Extensive purification to remove salting-out agent Possible incompatibility of salting out agents and drugs
Supercritical fluids-based technology	Environmentally friendly solvents Few traces of solvent in the final product	Limited by polymer solubility in the supercritical fluid Difficult to control particle size and morphology;
Spray drying	Residual organic phase is immediately evaporated Easy to set up	It is difficult to control drug distribution into the nanoparticles Adhesion of nanoparticles to the inner walls of spray dryer
Melting techniques	No solvents required	Not suitable for thermally-sensitive compounds (e.g., proteins) Many steps are required
*In situ* forming techniques	No need to recover particles	Solvent toxicity must be previously investigated.

As mentioned in the previous sections, several parameters are involved in process optimization and strongly influence the final particle size distribution. The most important degrees of freedom, as well as their influence on the final outcome are summarized in Table 7.

**Table 7:** Process variables and their effect on particle size.

**Process variable**	**Effect on average particle size**
Solvent	It depends on the specific solvent, i.e., its effect on emulsification.
Surfactant/stabilizer	It depends on the chemical nature of the stabilizer (ionic/non-ionic).
Shear rate	High shear rate decreases particle size.
PLA molecular weight	Size increases as molecular weight increases (the viscosity of dispersed phase increases).
PLA concentration	Size increases as polymer concentration increases (the viscosity of dispersed phase increases).
Stabilizer concentration	High stabilizer concentration (3% w/v or higher) decreases particle size.
Viscosity of the dispersed phase	Size increases as viscosity increases.

## The New Paradigm Introduced by Nanoparticles

While devices at macroscale (suture treads, polymer-coated stents, bone fixation screws, etc.) remain at the implantation site, nanoparticles, because of their size, are able to spread all over the body and to penetrate into cells. This introduces a new paradigm in the engineering of polymeric nanocarriers, since they must be designed so that they remain in the systemic circulation long enough to accomplish their task and they are able to target the desired objective. Particle behavior, in terms of clearance, biodistribution (i.e., distribution in the organs), cellular uptake, and toxicity are mainly influenced by particle size, shape, morphology, surface chemistry, and charge (Blanco et al., 2015). The techniques that can be used to characterize experimentally nanoparticles are summarized in Table 8 (Crucho and Barros, 2017).

**Table 8:** Experimental techniques for nanoparticles characterization (Crucho and Barros, 2017).

**Experimental technique**	**Nanoparticle property**
Atomic Force Microscopy	• Size and size distribution • Shape • Structure • Aggregation • Surface properties
Differential scanning calorimetry	• Physicochemical state and possible interactions between drug and polymer
Dynamic light scattering	• Particle size distribution (hydrodynamic radius);
Fluorescence microscopy	• Critical association concentration • Drug content • *In vitro* drug release
High performance liquid chromatography	• Drug content • *In vitro* drug release
Infrared spectroscopy	• Structure and conformation of bioconjugates • Functional group analysis
Mass spectrometry	• Molecular weight • Composition • Structure • Surface properties
Near-field scanning optical microscopy	• Size • Shape
Nuclear magnetic resonance	• Structure • Composition • Purity • Conformational change
Scanning electron microscopy	• Size and particle size distribution • Shape • Aggregation
Transmission electron microscopy	• Size and particle size distribution • Shape heterogeneity • Aggregation
X-ray photoelectron spectroscopy	• Elemental and chemical composition a the surface
Zeta potential	• Stability referring to surface charge

The acquired knowledge led to the development of proper design strategies, as shown below (*vide infra*). Nanoparticles synthesized from PLA-based materials are mainly employed as devices for drug delivery for cancer treatment and for imaging purposes (Kim et al., 2019). Nanoparticles are potentially able to penetrate selectively within the cancer, where they can release the loaded active compound at the desired rate, so that a therapeutic effective drug concentration is maintained for a given time period. This allows minimizing the amount of administered drug, since it mainly diffuses in the tumor following nanoparticles permeation through cancer cells, dampening potential side effects and optimizing costs for health systems.

There are various administration routes for nanoparticles, such as oral, parenteral (intravenous, subcutaneous, intradermal, and intramuscular), respiratory, and transdermal routes (Kaialy and Al Shafiee, 2016). In any case, nanoparticles must be able to cross certain barriers (which can vary according to the administration route) in order to be effective (Blanco et al., 2015). Depending on the administration route, the first barrier can be constituted by endothelial or epithelial cells.

Epithelium is essentially constituted by the skin and mucosal membranes, while endothelium separates the blood flow from the surrounding tissues. The endothelium that separates blood vessel and central nervous system is the well-known blood brain barrier (BBB), which is very challenging to cross.

Another barrier is constituted by the immune system; after injection, nanoparticles experience opsonization, which involves the adsorption of plasma proteins on the surface of the device that leads to the formation of the protein corona (*vide infra*). After the attainment of the layer of adsorbed proteins, nanoparticles bind to a macrophage receptor and are subsequently internalized and removed from circulation. This problem can be overcome through surface modification, hindering protein adsorption, and interactions with macrophages receptors. The most popular route is PEGylation, that is, the addition of PEG brushes on nanoparticle surface that constitute an obstacle for protein adsorption (Partikel et al., 2019). Other strategies involve surface functionalization with *ad hoc* peptides that delay phagocytic clearance (Rodriguez et al., 2013), or coating with cell membranes from red blood cells or leukocytes (Blanco et al., 2015). In general, the objective is to prolong the persistence in the blood circulation avoiding a rapid clearance by the immune system.

Focusing on PEGylation of PLA-based particles, three main approaches can be identified (Betancourt et al., 2009). In direct conjugation, PEG chains are covalently bound to the end groups of polymer chains already assembled in nanoparticles. This approach has the advantage that PEGylation is performed after the encapsulation of an active principle with standard techniques but it is not very efficient because of the limited exposure of the end groups on particle surface. When active conjugation in solution is chosen, preformed long polymer chains are activated and conjugated with PEG chains. Despite the moderate conjugation efficiency, the attainment of high yields is hindered by a difficult recover of the copolymer and the possible formation of PEG-PEG conjugates that can affect the purity of the product. The most used technique is ring opening polymerization, where preformed OH-PEG-COOH chains (that is, with a hydroxyl and a carboxyl end groups) are polymerized with lactide (and glycolide, if PLGA is needed) cyclic dimers. In these conditions, the hydroxyl end group of PEG acts as a protic agent initiating the reaction, while carboxyl end group remains intact. This leads to the synthesis of block PLA-PEG block copolymers.

Eventually, nanoparticles can experience cellular uptake mainly through endocytosis, which can be due to different pathways (Sahay et al., 2010; Kim et al., 2019). In receptor-mediated endocytosis, nanoparticles can be internalized by interacting with a specific receptor expressed on cellular membrane; a key role is played by clathrin and caveolin. Clathrin-mediated endocytosis is present in essentially all mammalian cells and is responsible for the uptake of essential nutrients; caveolae-mediated endocytosis exploits the presence of caveolin proteins in the caveolae (lipid rafts along cellular membranes) and attracted some interest since this pathway allows bypassing lysosomes and thus avoiding lysosomal degradation. Carrier-mediated endocytosis exploits the presence of carrier proteins on cellular membrane; this pathway can be exploited to pass challenging barriers like the BBB. Since the cellular membrane, at physiological pH, has a slight negative charge, electrostatic interactions with positively charged carriers can promote particle internalization through an adsorption-mediated endocytosis. Pinocytosis implies the formation of membrane-based vesicles from the cell surface, which captures solute and fluid from the environment.

From an experimental point of view, it is possible to identify the specific endocytosis pathway by suppressing some mechanisms with suitable inhibitors and assessing the cellular uptake.

In this regard, it useful to introduce the concept of targeting. In order to maximize their effect, nanoparticles should be able to selectively penetrate within the tumor, minimizing their accumulation in healthy organs. There are two different targeting approaches: passive and active targeting.

Passive targeting exploits the so called enhanced permeation and retention (EPR) effect; according to EPR, cancer exhibits an enhanced permeation due to the hyperpermeable vasculature and an enhanced retention because of the ineffective lymphatic drainage. Although EPR concept seems to be quite well-assessed in literature, its effectiveness is still debated since it is well-documented for small animal models but human clinical data are less clear (Danhier, 2016).

Active targeting implies the functionalization of nanoparticle surface with suitable ligands (small molecules, proteins, carbohydrates, etc.), which can interact in a specific way with receptors that are overexpressed in diseased organs, tissues and cells (Bertrand et al., 2014). Since PLA has no reactive side groups, functionalization needs the synthesis of polymer chains with end groups that can be activated for further conjugation. In this regard, two strategies can be identified; pre-conjugation, where conjugated chains are obtained and subsequently assembled in nanoparticles with a suitable technique. This approach can be used for small ligands and peptides, while it is not suitable for proteins, since they can affect self-assembling process and conjugation needs organic solvents that can influence the secondary structure. Pre-conjugation allows introducing multiple ligands with one-step formulation procedure and a good control of particle properties. Post-conjugation involves the functionalization of preformed nanoparticles; this strategy is suitable for both small and big ligands (proteins, antibodies).

Notably, functionalization can be achieved also through the physical (that is, non-covalent) adsorption of targeting moieties on nanoparticle surface (Bertrand et al., 2014).

Summarizing, Dawidczyk et al. (2014) proposed general guidelines for the design of nanoparticles as carriers of active compounds, as shown in Table 9.

**Table 9:** Design criteria for nanoparticles for drug delivery purposes (Dawidczyk et al., 2014).

**Function**	**Design requirements**	**Possible strategies**
Circulation	• Stable under flow at 37°C	• Avoid binding with components of blood • Neutral or slightly negative zeta potential
Distribution	• Minimize tissue (peripheral) volume • Minimize binding to endothelium • Minimize paracellular transport	
Elimination	• Minimize opsonization • Minimize recognition by phagocytic cells • Maximize circulation time • Minimize rapid clearance by the kidneys	• Stealth coating • diameter > 8 mm to avoid rapid clearance in kidneys
Tumor accumulation	• Maximize extravasation across tumor vasculature	• Diameter <200 nm for transport across leaky vasculature through EPR • Maintain high plasma concentration • Enhance EPR effect
Tumor cell uptake	• Maximize binding/uptake by tumor cells • Trafficking to cellular compartment	• Active or passive drug release at tumor site • Maximize cell death/particle • Maximize dose/particle • Maximize endosomal escape for particles taken up by endocytosis

In the following paragraphs, some relevant examples from scientific literature are reported concerning PLA-based nanoparticles for drug delivery and imaging purposes. Given the extent of the topic, the following discussion does not claim to be exhaustive, but aims at presenting some opportunities in the field.

### Nanoparticles for Drug Delivery

Shalgunov et al. (2017) systematically investigated the effect of PEG coverage, injected dose and release kinetics on the performance of PLA-PEG nanoparticles loaded with vincristine (an anticancer active compound), determining their impact on pharmacokinetics and biodistribution in *in vivo* animal model.

Pavot et al. (2013) synthesized PLA nanoparticles containing Nod1 and Nod2 receptors ligand; the aim is to induce a systemic immune response to improve the efficacy of vaccine delivery applications. Experimental outcomes showed promising results.

Zhou et al. (2018) developed nanoparticles based on hydroxyehyl starch-polylactide (HES-PLA) polymer, where they loaded two active compounds: doxorubicin and the TGF-β inhibitor LY2157299. This strategy involving combined delivery aims at suppressing both tumor growth and metastasis.

Medel et al. (2017) developed PLA-PEG nanoparticles loaded with anticancer drugs curcumin and bortezomib. These active compounds are highly hydrophobic and show synergistic effects; in addition, they can form a covalent complex stable at physiological pH but labile at midly acidic pH (such as cancer microenvironment). The use of nanoparticles improved the cytotoxicity provided by curcumin-bortezomib if compared to free, not-encapsulated drugs.

Raudszus et al. (2018) synthesized PLA nanoparticles using a newly developed stabilizer, a vinyl sulphone-modified poly(vinyl alcohol) (VS-PVA) derivative. By virtue of its enhanced reactivity, VS-PVA derivative allowed an easy functionalization of particle surface with targeting moieties such as Ovalbumin, Apolipoprotein E (ApoE), and Penetratin. In particular, ApoE and Penetratin functionalized particles exhibited a higher cellular uptake, associated to a specific interactions with cellular receptors.

Zhu et al. (2016) developed nanoparticles made of D-α-tocopherol polyethylene glycol succinate-poly(lactide) (TPGS-PLA) loaded with docetaxel, an anticancer compound. Nanoparticles were coated with polydopamine and functionalized with glucosamine in order to enhance cellular uptake in the liver through ligand-mediated endocytosis.

Zhang et al. (2016) synthesized PLA-PEG nanoparticles loaded with paclitaxel and functionalized with EGFP-EGF1 covalently bound to PEG brushes. *In vivo* experiments showed that such particles are able to target multiple types of key cells in tumor tissues.

Turino et al. (2017) developed paclitaxed-loaded PLGA nanoparticles functionalized with ferritin. Functionalization was possible thanks to the use of PLGA-NHS polymer, where one end group is constituted by succinimidyl ester, which reacts with protein amine groups. Nanoparticles were also loaded with a guanidinium-based (Gd-DOTAMA) agent for magnetic resonance imaging (MRI). Experimental studies *in vitro* proved the targeting capability using breast cancer cell lines.

Gourdon et al. (2017) investigated PLA-PEG nanoparticles loaded with acyclovir (antiviral drug) and functionalized with single amino acids or short peptides in order to target PepT1 intestinal transporter. Functionalization was performed by covalently linking amino acids to PEG chains with an amine end group. Valine-functionalized nanoparticles showed the best outcomes in terms of targeting.

Cui and Zhu (2016) prepared doxorubicin-loaded PLA nanoparticles, covered with polyethylene imine (PEI) that was functionalized with Herceptin, a monoclonal antibody, which targets the human epidermal growth factor receptor 2 (HER2), overexpressed in breast cancer. Functionalization improved cellular uptake and nanoparticles proved to enhance the therapeutic effect of the drug reducing side effects.

Xiong et al. (2016) synthesized a block copolymer containing folic acid, pluronic (a polyethylene oxide-poly propylene oxide-polyethylene oxide block copolymer) and lactic acid. Resulting product was employed to produce paclitaxel-loaded nanoparticles. Experimental data proved that folate included in polymer chain could be used for active targeting with folate receptor expressed in ovarian cancer cells.

Coolen et al. (2019) synthesized PLA nanoparticles, which exhibit a negative charge on the surface due to lactic acid resulting from degradation. In order to non-covalently bind mRNA on the surface (they are both negatively charged), the authors firstly created a non-covalent complex between mRNA and cationic cell penetrating peptides (CPPs), which could be adsorbed on PLA surface.

Tang et al. (2018) obtained PLA-PEG micelles loaded with paclitaxel, an anticancer drug. They functionalized the surface with a CPP linked to PEG polymer with a pH-sensitive sequence composed of histidine and glutamic acid. At physiological pH, CPP, and the linker are strongly bound through electrostatic interactions between glutamic acid (in the linker) and arginine (in the CPP). The mildly acidic pH of the tumor microenvironment leads to the protonation of some histidine residues of the linker, which interfere with the linker/CPP electrostatic interactions. The immediate consequence is an increased exposure of the CPP in the cancer and thus a more effective targeting, which could be achieved with stimuli responsive device (pH, in this case).

Song et al. (2016) developed PLA-PEG nanoparticles loaded with AZD2811, a hydrophobic anticancer active compound. The authors extensively tested the hydrophobic ion pairing (HIP) approach in order to maximize drug loading and encapsulation efficiency. They evaluated different hydrophobic counterions (such as oleic acid, cholic acid, and so on) that increase AZD2811 hydrophobicity through the formation of ion pairs. Different counterions led to different release kinetics, which allowed obtaining a library of particle formulations.

Medina et al. (2019) synthesized PLA-PEG and PLGA nanoparticles, blended with low molecular weight PLA and PCL and lipid-conjugate PEG, respectively. They observed that this blending improved the encapsulation efficiency of adapalene (a topical retinoid). Blending had a moderate impact on release kinetics.

Discussed examples are summarized in Table 10.

**Table 10:** Summary of discussed examples of nanoparticles for drug delivery.

**System**	**Preparation method**	**Average size [nm]**	**Loaded compounds**	**Main features**	***In vivo***	**References**
PLA-PEG	Single emulsion	100	Vincristine	Systematic investigation of PEG coverage, release kinetics and injected dose	Yes	Shalgunov et al., 2017
PLA	Nanoprecipitation	200	Nod receptor ligands	Induced systemic immune response for vaccine delivery	Yes	Pavot et al., 2013
HES-PLA	Single emulsion	155	Doxorubicin and TGF-β inhibitor LY2157299	Co-delivery of two active compounds	Yes	Zhou et al., 2018
PLA-PEG	Nanoprecipitation	100–150	Curcumin, curcumin and bortezomib	Synergistic effects with co-delivery	No	Medel et al., 2017
PLA-VS-PVA	Double emulsion	220	Lumogen Red (dye)	New stabilizer for easier surface functionalization	No	Raudszus et al., 2018
TPGS-PLA	Single emulsion	200	Docetaxel	Nanoparticles coated with polydopamine and functionalized with galactosamine	Yes	Zhu et al., 2016
PLA-PEG	Single emulsion	100–120	Paclitaxel	Surface functionalization with EGFP-EGF1 protein to enhance active targeting	Yes	Zhang et al., 2016
PLGA-PEG	Single emulsion	150	Paclitaxel	Surface functionalization with ferritin for targeting, Gd-DOTAMA as imaging agent	No	Turino et al., 2017
PLA-PEG	Nanoprecipitation	30–50	Acyclovir	Surface functionalization with amino acids	Yes	Gourdon et al., 2017
PLA-PEI	Single emulsion	140–220	Doxorubicin hydrochloride	Surface functionalization with antibodies	Yes	Cui and Zhu, 2016
FA-Pluronic-PLA	Dialysis	190–260	Paclitaxel	Synthesis of block copolymer with folate groups that target folate receptors	Yes	Xiong et al., 2016
PLA	Nanoprecipitation	200–240	mRNA	mRNA adsorbed on surface through electrostatic interactions	No	Coolen et al., 2019
PLA-PEG micelles	Thin-film hydration	25	Paclitaxel	pH-responsive system	Yes	Tang et al., 2018
PLA-PEG	Single emulsion	90–130	AZD2811	Development of a formulation library with different release kinetics	Yes	Song et al., 2016
PLA-PEG PLGA	Single emulsion	115–130	Adapalene	Blending with short chains of aliphatic polyesters or lipid improves encapsulation	Yes	Medina et al., 2019

### Nanoparticles for Imaging

Banerjee et al. (2017) radiolabeled with ^111^In-containing moieties and IRDye680RD PLA-PEG nanoparticles functionalized with prostate-specific membrane antigen (PSMA) moieties. *In vivo* and *ex vivo* imaging allowed determining the distribution of both PSMA-functionalized and not-functionalized particles in the tumor. Xiong et al. (2017) treated Fe_3_O_4_ iron oxide nanoparticles with PLA-PEG chains for MRI purposes. End groups of PLA-PEG chains where functionalized with D-glucosamine as targeting agent. *In vivo* MRI in tumor-bearing mice confirmed the ability of the nanoparticles to target the cancer and their potential as contrast agents.

Dos Santos et al. (2017) synthesized PLA nanoparticles loaded with betamethasone and dexamethasone (antiflammatory drugs) and labeled with technetium-99m. Experiments showed that betamethasone loaded particles were able to accumulate in the inflammation site in an *in vivo* model of *S. aureus* infection.

Kerr et al. (2017) synthesized dye-PLLA-conjugates using Difluoroboron β-Diketonates as dyes, which were subsequently employed to obtain nanoparticles (average diameter 55 nm) for imaging purposes. In order to improve stability, the authors added PDLA-PEG to promote stereocomplexation. *In vivo* experiments proved the ability of such particles to target tumors.

## Toxicity of Polylactic-Based Nanoparticles

Nanoparticles behavior mainly depends on size, shape, morphology, and surface charge; this holds also for their unwanted side effects. Entering into cells, nanoparticles can provide cytotoxic effects, leading to the disruption of cell membranes or cytosolic components or to programmed cell death (apoptosis). Typical adverse effects are oxidative stress [an excess of reactive oxygen species (ROS) or reactive nitrogen species (RNS) usually neutralized by cells], apoptosis, cytokine activation (due to inflammatory response), loss of mitochondrial, and lysosomal stability. They can also be a source of genotoxic effects, damaging DNA (Ganguly et al., 2018).

In addition, nanoparticles may induce haemolysis (disruption of red blood cells) or blood coagulation (causing thrombosis) (Dobrovolskaia and McNeil, 2007). PLA-based nanoparticles may provide additional side effects through their degradation products.

Toxicity assessment *in vitro* in usually performed by exposing cells to given dose of the potential toxic agent and measuring, e.g., cell viability and proliferation, mitochondrial activity, ROS production, cytokine activation through suitable assays.

*In vivo* experiments aim at assessing the pharmacokinetics of nanoparticles, their distribution in the organ and their clearance.

While *in vitro* and *in vivo* testing are usually performed when a new formulation is discussed (*vide* supra) up to author's best knowledge, there are few systematic studies concerning toxicity of PLA-based nanoparticles (Da Luz et al., 2017; Da Silva et al., 2019).

Singh and Ramarao (2013), e.g., observed that PLA nanoparticles *in vitro* did not provide detrimental effect concerning RNS, cytokine activation, mitochondrial or lysosomal integrity. At high concentration, they stimulated ROS production and inflammation; this was linked to the accumulation of polymer degradation products in the cell.

There is an additional intrinsic risk of toxic responses when nanoparticles are used and injected in the blood stream. Nanoparticles interact with the components present in the environment (proteins, carbohydrates, small molecules, etc.) through their surface. The driving force leading to the formation of this nano-bio interface are already known in scientific literature and are basically due to electrostatic and Van der Waals interactions as well as hydrophobic and depletion effects (Nel et al., 2009). One of the main outcomes from this network of interactions is the attainment of a layer of adsorbed proteins on nanoparticle surface, usually referred as protein corona (Cedervall et al., 2007). Because of the interactions with the surface, adsorbed proteins can be subjected to relevant structural changes, which can lead to aggregation and fibrillation, loss of enzymatic activity, or the exposure of new antigenic epitopes. Such side effects emerge from the specific protein-surface interactions: while, as mentioned, driving forces are known, they depend on many factors (materials, pH, ionic strength, etc.) and are challenging to be determined *a priori*.

*In vitro* experiments allow a rapid and cost-effective evaluation of toxicity if compared to *in vivo* experiments with animal models (and the related ethical concerns). However, the possible lack of correlation between *in vitro*-*in vivo* tests, the challenging extrapolation of animal data to human patients and the shortage of harmonized protocols are still obstacles for extensive clinical trials (Dobrovolskaia and McNeil, 2013).

## Conclusions

PLA—based polymers have been extensively studied in literature and are currently an established reality in the biomedical field, thanks to their interesting properties. This led to a growing interest also in the nanomedicine field for the synthesis of nanoparticles for drug delivery and imaging purposes. Nanoparticles showed a great potential as nanocarriers to deliver poorly soluble drugs, proteins, and genes targeting the tumor and releasing the active compound at the desired rate, enhancing in the therapeutic effect.

The new perspective introduced by nanoparticles also brings new sources of toxicity connected with cytotoxicity and haemolysis; also protein corona can provide undesired side effects that are not easily predictable *a priori*.

Nowadays, an extensive clinical application of nanoparticles is still hindered by an exhaustive assessment of potential toxic effects, which does not allow nanoparticles unleashing their full potential.

## Author Contributions

TC performed literature research and wrote the first draft of the paper. All authors discussed and approved the contents of the manuscript and contributed to its final version by reading and editing.

### Conflict of Interest

The authors declare that the research was conducted in the absence of any commercial or financial relationships that could be construed as a potential conflict of interest.
